# Supplementary material for: Indicators to evaluate the labor insertion of people with disabilities in conventional companies in Spain: A Delphi study
Source: PLoS One. 2025 May 9;20(5):e0322814. doi: 10.1371/journal.pone.0322814 (PMC12063877; doi:10.1371/journal.pone.0322814)
Supplement: Table S1 — (DOCX) [file pone.0322814.s001.docx]

**Supporting information**

**Table S1. Comparation of the scores of the two types of experts in third phase.**

| Selection and Recrutiment | Conventional companies | | Third sector | | p-value |
| --- | --- | --- | --- | --- | --- |
| SR1 Adapt recruitment process | Median | IQR | Median | IQR | 0.007 |
| SR2 Adapt selection | 8.5 | 2.75 | 10 | 1 | 0.021 |
| SR3 Score pwD on training | 9 | 2.75 | 10 | 1.75 | 0.23 |
| SR4 Score pwD work experience | 7 | 2 | 8 | 2.75 | 0.152 |
| SR5 Score pwD on soft skills | 7 | 2 | 8 | 2 | 0.309 |
| SR6 Score pwD on job-specific technical competencies. | 8 | 1.75 | 8 | 1.75 | 0.328 |
| SR7 Score pwD digital competencies | 8 | 1 | 8 | 2 | 0.268 |
| SR8 Assess the external support | 7 | 1.75 | 7 | 2.62 | 0.055 |
| SR9 Define job characteristics | 5 | 2 | 7 | 3 | 0.062 |
| SR10 Have people trained in disability | 9 | 1 | 10 | 0.75 | 0.002 |
| SR11 Have advice from external specialists in labor insertion of pwD. | 8 | 3.75 | 9 | 2 | 0.07 |
| SR12 Rate of hiring of pwD | 8.5 | 2 | 9 | 2 | 0.311 |
| SR13 % of candidates with disabilities in selection process | 7 | 2.75 | 6.75 | 3.75 | 0.914 |
| Work Itinerary | Conventional companies | | Third Sector | | p-value |
|  | Median | IQR | Median | IQR |  |
| WI1 Adapted welcome plan | 9 | 2 | 10 | 1 | 0.049 |
| WI2 Adapted training and professional development itineraries | 8 | 2 | 10 | 1 | 0 |
| WI3 Implement career plans for the pwD | 7 | 2.25 | 9 | 2 | 0 |
| WI4 Specialized internal mentor that accompanies the pwD | 8 | 3 | 9 | 2 | 0.006 |
| WI5. PwD retention rate. | 8 | 1.75 | 8.5 | 3 | 0.032 |
| WI6 Promotion opportunities for the pwD | 7.5 | 2.5 | 8.5 | 2.75 | 0.051 |
| WI7 Mindset of pwD | 8 | 2 | 9 | 2 | 0.028 |
| Work Performance | Conventional companies | | Third sector | | p-value |
|  | Median | IQR | Median | IQR |  |
| WP1 Work performance according to company parameters | 8 | 2 | 8 | 2 | 0.727 |
| WP2 pwD productivity | 8 | 2 | 8 | 1.75 | 0.333 |
| WP3 Performance of pwD | 8 | 2 | 9 | 2 | 0.038 |
| WP4 Workloads of inclusive teams. | 8 | 2 | 9 | 2 | 0.032 |
| WP5 Work rhythms in inclusive teams. | 8 | 2 | 9 | 2 | 0.034 |
| WP6 Quality of the work of pwD. | 8 | 2 | 9 | 2 | 0.098 |
| WP7 Fulfillment of objectives by pwD. | 9 | 1 | 9 | 2 | 0.267 |
| WP8 Participation of pwD in projects | 9 | 1 | 9 | 2 | 0.419 |
| WP9 Participation of pwD in meetings. | 8 | 2 | 9 | 2 | 0.073 |
| WP10 Involvement of pwD in problem solving | 8 | 2 | 9 | 2 | 0.044 |
| WP11 pwD absenteeism | 8 | 2.5 | 9 | 2 | 0.046 |
| WP12 pwD punctuality | 8 | 3 | 9 | 2 | 0.017 |
| WP13 pwD Interaction with colleagues | 9 | 2.5 | 8 | 1.75 | 0.878 |
| WP14 pwD empathy and assertiveness | 7 | 2 | 8 | 2.75 | 0.198 |
| WP15 Job task autonomy of the pwD. | 8 | 1 | 9 | 2 | 0.3 |
| WP16 company flexibility for incorporation of pwD | 8 | 2.75 | 9 | 2 | 0.044 |
| WP17 Accesible complaints and claims | 9 | 1.75 | 9 | 1 | 0.113 |
| Work Environment | Convencional companies | | Third sector | | p-value |
|  | Median | IQR | Median | IQR |  |
| WE1 Perception of colleagues associated with the incorporation of pwD | 8 | 2 | 8.5 | 2 | 0.506 |
| WE2 Training needs regarding disability in the company | 8 | 1 | 9 | 2 | 0.05 |
| WE3 Impact on fellowship for the incorporation of pwD | 8 | 1.75 | 9 | 1.75 | 0.904 |
| WE4 Improvement of the team's self-esteem for incorporation of pwD | 8 | 2 | 9 | 1 | 0.426 |
| WE5 Impact on innovation in team for the incorporation of pwD | 7 | 1.75 | 9 | 1.75 | 0.019 |
| WE6 Team's empathy with pwD | 9 | 1 | 9 | 2 | 0.264 |
| WE7 Training of work team in disability | 9 | 2 | 9 | 2 | 0.574 |
| WE8 Perception of the pwD in relation to feelings of acceptance for colleagues | 9 | 1.75 | 9 | 1.75 | 0.186 |
| Socially Responsible Company | Convencional companies | | Third sector | | P-value |
|  | Median | IQR | Median | IQR |  |
| SRC1 Reputation benefits when incorporating PwD | 8 | 1.75 | 8 | 2 | 0.673 |
| SRC2 Accessibility of the environment | 8.5 | 2 | 10 | 1 | 0.01 |
| SRC3 Importance for the workforce of having an accessible environment | 8.5 | 2 | 9.5 | 1.75 | 0.009 |
| SRC4 Benefits obtained by pwD for working | 8 | 1.75 | 9 | 2 | 0.115 |
| SRC5 Company benefits by incorporating PwD | 8 | 2 | 9 | 2 | 0.131 |
| SRC6 Cost of adaptation and support measures and actions for the pwD. | 7.5 | 1.75 | 8 | 2.75 | 0.082 |
| SRC7 Mindset of the teams for working in an inclusive company. | 8 | 1.75 | 9 | 2 | 0.047 |

It is shown the median, interquartile range (IQR), and p-value of Wilcoxon test. SR: Selection and Recruitmen; WI: Work Itinerary; WP: work Performance; WE: Work Environment; SRC: Socially Responsible Company.
