# Supplementary material for: Indicators to evaluate the labor insertion of people with disabilities in conventional companies in Spain: A Delphi study
Source: PLoS One. 2025 May 9;20(5):e0322814. doi: 10.1371/journal.pone.0322814 (PMC12063877; doi:10.1371/journal.pone.0322814)
Supplement: Attachment — Submitted filename: Response to reviewers v-2.docx [file pone.0322814.s003.docx]

REBUTTAL LETTER

Contenido

[EDITOR´S COMMENTS1](#_Toc188182238)

[REVIEWER 1	6](#_Toc188182239)

[REVIEWER 2	27](#_Toc188182240)

[Review of manuscript "INDICATORS TO EVALUATE THE LABOUR INSERTION OF PEOPLE WITH DISABILITIES IN CONVENTIONAL COMPANIES IN SPAIN: A DELPHI STUDY."	37](#_Toc188182241)

First of all, we appreciate the effort and time invested in this manuscript. These are very valuable comments that we are sure, they enhance its quality.

We will proceed to respond point by point to the comments from the editor and reviewers.

# **EDITOR´S COMMENTS**

**A review of bibliographic references is required**.

Thank you for your comments. A review of the bibliographic references has been done, and some references has been added:

[3] World Health Organization (WHO). World report on disability. 2011. Available from: <https://www.who.int/es/publications/i/item/9789241564182>

[4] Fullana Noell, J. F., Díaz, M. P., & Suñé, M. V. (2003). [Research on the labor integration processes of people with disabilities in ordinary settings. A qualitative case study]. Revista de Investigación Educativa. 2003; 21(2): 305-321. Available from: [<http://riberdis.cedid.es/bitstream/handle/11181/3369/Investigacion_sobre_procesos_integracion_laboral_personas_con_discapacidad.pdf>]

[5] State Public Employment Service (SEPE). [Report on the labor market for people with disabilities. Spain. Data 2023.]. 2024. Available from: [<https://sid-inico.usal.es/wp-content/uploads/2024/04/Informe-del-Mercado-de-Trabajo-2023.pdf>]

[7] Royal Board on Disability (RPsD), Ministry of Labor and Social Economy, CERMI, & ONCE Foundation. [White Paper on Employment and Disability]. 2023. Available from: [<https://www.rpdiscapacidad.gob.es/estudios-publicaciones/libro_blanco_empleo_discapacidad.htm>]

[9] Ishikawa, K., & Loftus, J. H. Introduction to quality control. Chapman & Hall. 1991.

[10] Peiró, JM. [Introduction to work psychology]. Madrid: Ediciones CEF. 2013

[16] Villa N. [Employment situation of people with disabilities in Spain]. Rev. complut. Educ. 2003;14(2):393-424

[19] Fundación ONCE & Fundación Manpower. [Study on the perspectives of the business world with respect to the hiring of people with disabilities]. Madrid: Fundación ONCE. 2008. Available from: [<https://consaludmental.org/publicaciones/Perspectivasempresarialcontratacionpersonasdiscapacidad.pdf>]

[21] Mira-Aladrén M, Yagüe-Sanjuán N, Latorre-Dena F. [Disability, inclusion and public employment, is it possible to improve their relationship? Reflections from a pilot experience at the University of Zaragoza]. Acciones e Investigación Sociales. 2024; (45). Available from: [<https://papiro.unizar.es/ojs/index.php/ais/article/view/9024>]

[26] Fundación ONCE. [Incentives for hiring people with disabilities]. Fundación ONCE. 2023. Available from: <https://www.portalento.es/IncentivosContratacionPCD.pdf>]

[27] Sánchez, A., Fernández, M.P., Pérez Nieto, M.A., Carpintero, E., Pérez Fernández, F. y Mampaso, J. [Study on the social and labor reality of people with disabilities in the Community of Madrid: analysis of the existing legislative measures of insertion and degree of compliance with them by companies]. Madrid: UCJC. 2008.

[29] Niederberger, M., Schifano, J., Deckert, S., Hirt, J., Homberg, A., Köberich, S., ... & DEWISS network. Delphi studies in social and health sciences—Recommendations for an interdisciplinary standardized reporting (DELPHISTAR). Results of a Delphi study. Plos one. 2024; 19(8): e0304651. [DOI: 10.1371/journal.pone.0304651](https://doi.org/10.1371/journal.pone.0304651)

[30] Von der Gracht, H. A. Consensus Measurement in Delphi Studies Review and Implications for Future Quality Assurance. Technological Forecasting & Social Change. 2012; 79(8): 1525–1536 (1986). DOI: 10.1016/j.techfore.2012.04.013

[31] Carl J, Mazzoli E, Mouton A, Sum RK-W, Singh A, Niederberger M, et al. (2024) Development of a Global Physical Literacy (GloPL) Action Framework: Study protocol for a consensus process. PLoS ONE. 2024; 19(8): e0307000. [DOI: 10.1371/journal.pone.0307000](https://doi.org/10.1371/journal.pone.0307000)

[32] Okoli, C., & Pawlowski, S. D. The Delphi method as a research tool: an example, design considerations and applications. Information & Management. 2004; 42(1),:15-29. DOI: 10.1016/j.im.2003.11.002

[33] Flostrand, A., Pitt, L., & Bridson, S. The Delphi technique in forecasting–A 42-year bibliographic analysis (1975–2017). Technological Forecasting and Social Change. 2020; 150: 119773. (157). [DOI: 10.1016/j.techfore.2019.119773](https://doi.org/10.1016/j.techfore.2019.119773)

[34] Dalkey, N., & Helmer, O. (1963). An experimental application of the Delphi method to the use of experts. *Management science*. 1963;  *9*(3): 458-467.

[36] Rowe, G., y Wright, G. The Delphi technique as a forecasting tool: issues and analysis. International Journal of Forecasting. 1999; 15(4): 353-375.

[37] Belton, I., MacDonald, A., Wright, G., & Hamlin, I. Improving the practical application of the Delphi method in group-based judgment: A six-step prescription for a well-founded and defensible process. Technological Forecasting and Social Change. 2019; 147: 72-82. [DOI: 10.1016/j.techfore.2019.07.002](https://doi.org/10.1016/j.techfore.2019.07.002)

[38] García-Uceda, E. y Murillo-Luna, JL [Application of the Delphi method for the analysis of the determinants of Social Entrepreneurship.]. Ponencia. XXVII Jornadas Hispano_Lusas de Gestión Científica. Localización y dinámicas competitivas en un entorno global. Libro de ACTAS/ Resúmenes. 2017. IBSN: 978-84-16724-37-6; 1 – 4 febrero. Benidorm (Alicante).

[40] Martínez E. The Delphi Technique as a stakeholder consultation strategy for programme evaluation. Revista de Investigación Educativa. 2003: 21(2): 449-163.

[41] García, L., Fernández, S. Procedimiento de aplicación del trabajo creativo en grupo de expertos. Energética, XXIX (2). 2008; 46-50.

[42] Devaney, L., & Henchion, M. (2018). Who is a Delphi ‘expert’? Reflections on a bioeconomy expert selection procedure from Ireland. Futures; 2018; 99: 45-55. DOI: 10.1016/J.FUTURES.2018.03.017.

[45] Boulkedid, R., Abdoul, H., Loustau, M., Sibony, O., & Alberti, C. (2011). Using and Reporting the Delphi Method for Selecting Healthcare Quality Indicators: A Systematic Review. PLoS ONE. 2011;6(6):e20476. doi: 10.1371/journal.pone.00204766.

[50] Landeta, J., y Lertxundi, A. Quality indicators for Delphi studies. Futures & Foresight Science. 2024; 6(1). e172. [DOI: 10.1002/ffo2.172](https://doi.org/10.1002/ffo2.172)

[51] Niederberger, M., & Spranger, J. Delphi Technique in Health Sciences: A Map. Frontiers in public health. 2020; 8: 457. DOI: 10.3389/fpubh.2020.00457.

[52] Skulmoski, G. J., Hartman, F. T., & Krahn, J. The Delphi method for graduate research. Journal of Information Technology Education: Research. 2007; 6(1), 1-21. DOI: 10.28945/199

[53] Niederberger, M., Köberich, S., & DeWiss Network. Coming to consensus: the Delphi technique. European Journal of Cardiovascular Nursing. 2021; 20: 692–695. DOI:10.1093/eurjcn/zvab059

[56] Valls, M. J., Vila, M & Pallisera, M. [The insertion of people with disabilities in ordinary work. The role of the family]. Revista de educación. 2004, 334(1): 97-117.

[61] Pico Barrionuevo, F. P., y Torres, S. S. [Best corporate social responsibility practices in the inclusion of people with disabilities. A case study of companies in Ambato, Ecuador]. Retos. 2017; 7(14). DOI: 10.17163/ret.n14.2017.10

[62] Pupiales, B. E., y Andrade, L. C. [The labor inclusion of people with disabilities: An ethnographic study in five autonomous communities in Spain.]. Archivos de Medicina (Manizales). 2016; 16(2): 279-289. DOI: 10.30554/ARCHMED.16.2.1720.2016

[66] Ortiz-Marcos, J. M., Invernón-Gómez, A. I., Medina-García, M., & Higueras-Rodríguez, L. [Labor inclusion of people with disabilities: Legislative challenges and practical solutions.]. Revista Española de Orientación y Psicopedagogía. 2024; 35(3): 105-122. [DOI: reop.vol.35.num.3.2024.40820](https://doi.org/10.5944/reop.vol.35.num.3.2024.40820)

[70] Kuznetsova, Y., Yalcin, B. Inclusion of persons with disabilities in mainstream employment: is it really all about the money? A case study of four large companies in Norway and Sweden. Disability & Society. 2017; 32(2): 233–253. DOI:10.1080/09687599.2017.1281794

[71] Shaw, J., Wickenden, M., Thompson, S., & Mader, P. Achieving disability inclusive employment – Are the current approaches deep enough? Journal of International Development. 2022; 34(5): 2022, 942–963. [DOI: 10.1002/jid.3692](https://doi.org/10.1002/jid.3692)

[72] Darcy, S., Taylor, T., & Green, J. “But I can do the job”: examining disability employment practice through human rights complaint cases. Disability & Society. 2016; 31(9): 1242–1274. [DOI: 10.1080/09687599.2016.1256807](https://doi.org/10.1080/09687599.2016.1256807)

[73] Betancourt, D. C., Echeverri, M. P. G., & Rubio, L. A. Working Inclusion: A Pending Commitment of Organizations towards People with Disabilities. Siglo Cero. 2024; 55(1): 49–65. DOI: 10.14201/scero.31335

[74] Pallisera, M., Vila, M., Valls, M. J., Rius, M., Fullama, J., Jiménez, P., Cardona, M., & Lobato, J. [The labor integration of people with disabilities in the ordinary company in Spain: a research approach.]. Revista Española sobre Discapacidad Intelectual. 2003; 34: 5–18.

[75] Shore, C., Wright, S., & Però, D. (Eds.). Policy worlds: Anthropology and the analysis of contemporary power (Vol. 14). Berghahn Books. 2011.

[76] Robinson, G. and Dechant, K. (1997) Building a Business Case for Diversity. Academy of Management Executive, 1997; 11: 21-31. [DOI: 10.5465/AME.1997.9709231661](https://doi.org/10.5465/AME.1997.9709231661)

[77] Herring, C. Does diversity pay?: Race, gender, and the business case for diversity. American sociological review. 2009; 74(2): 208-224. DOI: 10.1177/000312240907400203

[78] Stone, D. L., & Colella, A. A model of factors affecting the treatment of disabled individuals in organizations. *Academy of management review. 1996*, *21*(2), 352-401.

[79] Booth, A., & Leigh, A. Do employers discriminate by gender? A field experiment in female-dominated occupations. Economics Letters. 2010; *107*(2): 236-238. [DOI: 10.1016/j.econlet.2010.01.034](https://doi.org/10.1016/j.econlet.2010.01.034)

[80] Lindsay, S., Osten, V., Rezai, M., & Bui, S. Disclosure and workplace accommodations for people with autism: a systematic review. Disability and rehabilitation. 2021; 43(5): 597–610. DOI: 10.1080/09638288.2019.1635658

[81] Chen A, O’Neill J, Phillips KG, Houtenville AJ, Katz E. Relationship of business practices and characteristics to supervisors’ perceived effectiveness of disability recruitment. Journal of Vocational Rehabilitation. 2023;59(3):301-310. DOI:10.3233/JVR-230047

[82] Rezai, M., Kolne, K., Bui, S. et al. Measures of Workplace Inclusion: A Systematic Review Using the COSMIN Methodology. J Occup Rehabil. 2020; 30: 420–454. [DOI: 10.1007/s10926-020-09872-4](https://doi.org/10.1007/s10926-020-09872-4)

[83] Adjo, J., Maybank, A., & Prakash, V. Building inclusive work environments. Pediatrics, 2021;148(Supplement 2). [DOI: 10.1542/peds.2021-051440E](https://doi.org/10.1542/peds.2021-051440E)

[84] Watkins, M. W. Exploratory factor analysis: A guide to best practice. Journal of black psychology. 2018; 44(3): 219-246. DOI: 10.1177/0095798418771807

In addition, the updating of contents has led to the elimination of some references, such as the following:

- International Labour Organization (ILO). World report on disability. 2020. Available from: [https://www.ilo.org/global/publications/books/WCMS_734617/lang--en/index.htm]
- Palomo R. [Factors influencing the employment rate of people with disabilities]. Informe Anual de Empleo, 2012, 14, 123-135.
- National Statistical Institute (INE). [Disabled People's Labour Market Report]. 2023. Available from: [https://www.ine.es/prensa/epd_2022.pdf]
- Taborda Morales, Y. L. T. [Business productivity and inclusion of people with disabilities: challenges and opportunities]. Revista Perspectiva Empresarial. 2023. 10(1), 3-5. [DOI: 10.16967/23898186.808](https://doi.org/10.16967/23898186.808)
- Barragán R. The inclusion of people with disabilities in the labour market: challenges and opportunities. Journal of Disability Policy Studies. 2017, 27(3): 131-140. DOI: 10.1177/1044207316656325

A reorganization of the citation in the manuscript has been performed.

# REVIEWER 1

**Interesting article in terms of its purpose and methodology, but it has limitations for publication, at least in its current version.**

**I list some weaknesses that should be improved, in my opinion, in order to be published in a journal of the quality of PLOS ONE**

Thank you very much for your comments. They have been very useful to improve the quality of the manuscript.

**Better justification of the need for this set of indicators: For whom are they needed. Who says they are needed. Why and what they are needed for. Why for PwD and not for all workers.**

The rationale for the study has been expanded in the revised version to address in greater detail the specific challenges faced by people with disabilities (PwD) in their access to and development in the labor market. These challenges include structural, cultural and attitudinal barriers that limit their employment opportunities and hinder their full integration into mainstream work environments. Labor inclusion not only implies access to employment, but also the creation of conditions for PwD to thrive and develop their potential.

The lack of comprehensive tools to assess the labor inclusion of PwDs beyond hiring rates has also been highlighted. Although these rates are important indicators, they do not capture key aspects such as organizational climate, accessibility and the impact of adaptations in the work environment. This shortcoming hinders the implementation of comprehensive strategies to promote inclusive work environments.

Finally, references to national and international policy frameworks that underline the relevance of inclusive practices adapted to the needs of PwD have been incorporated. Documents such as the UN Convention on the Rights of Persons with Disabilities, the European Disability Strategy 2021-2030 and the White Paper on Employment and Disability (Spain) provide clear guidelines for the promotion of labor inclusion provide clear guidelines for the promotion of labor inclusion. These frameworks emphasize the need to go beyond general diversity measures by proposing specific approaches to ensure equal participation of PwDs in the labor market.

The following has been added:

Lines 45-54: However, measuring success in workplace inclusion remains a challenge, due to the lack of specific tools that assess both individual adaptations and organizational practices. This issue not only responds to an ethical and human rights imperative [1], but also offers specific benefits for businesses and the economy in general [2]. According to the International Report on Disability, around one billion people, or 15% of the world's population, live with some form of disability and many of them face significant barriers to access formal employment [3]. In this context, having specific indicators would facilitate the objective evaluation of inclusive policies and practices, allowing companies to measure their impact and improve their strategies.

Lines 59-60: In Spain, people with disabilities represent 6.33% of the working-age population, but their activity rate is significantly lower, reaching 35.3% in 2023 [5]. This gap highlights the need for instruments to identify not only the existing barriers, but also the actions required to overcome them and evaluate their effectiveness.

Lines 65-67: In Spain, the White Paper on Employment and Disability proposes a legislative and public policy framework for the employment and right to work of people with disabilities [7].

Lines 71-74: The development of specific indicators that go beyond hiring rates and are adapted to the characteristics of PwD is crucial to evaluate the success of their inclusion in the regular company as well as to encourage the implementation of inclusive practices [5,7].

**Methodology:
Correct choice of technique, but: better justification for the choice of this technique (and not another). Supporting literature on the choice.**

Additional literature has been incorporated to strengthen the methodological justification, including references, which underline the Delphi method’s appropriateness for addressing complex, multidisciplinary problems with limited empirical evidence.

**Delphi literature additional to that presented, more up-to-date. Use of references from authors with more contrasted expertise in this methodology (see some references at the end of the review)**

Some references have been cited and included in the text.

[29] Niederberger, M., Schifano, J., Deckert, S., Hirt, J., Homberg, A., Köberich, S., ... & DEWISS network. Delphi studies in social and health sciences—Recommendations for an interdisciplinary standardized reporting (DELPHISTAR). Results of a Delphi study. Plos one. 2024; 19(8): e0304651. [DOI: 10.1371/journal.pone.0304651](https://doi.org/10.1371/journal.pone.0304651)

[30] Von der Gracht, H. A. Consensus Measurement in Delphi Studies Review and Implications for Future Quality Assurance. Technological Forecasting & Social Change. 2012; 79(8): 1525–1536 (1986). DOI: 10.1016/j.techfore.2012.04.013

[31] Carl J, Mazzoli E, Mouton A, Sum RK-W, Singh A, Niederberger M, et al. (2024) Development of a Global Physical Literacy (GloPL) Action Framework: Study protocol for a consensus process. PLoS ONE. 2024; 19(8): e0307000. [DOI: 10.1371/journal.pone.0307000](https://doi.org/10.1371/journal.pone.0307000)

[32] Okoli, C., & Pawlowski, S. D. The Delphi method as a research tool: an example, design considerations and applications. Information & Management. 2004; 42(1),:15-29. DOI: 10.1016/j.im.2003.11.002

[33] Flostrand, A., Pitt, L., & Bridson, S. The Delphi technique in forecasting–A 42-year bibliographic analysis (1975–2017). Technological Forecasting and Social Change. 2020; 150: 119773. (157). [DOI: 10.1016/j.techfore.2019.119773](https://doi.org/10.1016/j.techfore.2019.119773)

[34] Dalkey, N., & Helmer, O. (1963). An experimental application of the Delphi method to the use of experts. *Management science*. 1963;  *9*(3): 458-467.

[36] Rowe, G., y Wright, G. The Delphi technique as a forecasting tool: issues and analysis. International Journal of Forecasting. 1999; 15(4): 353-375.

[37] Belton, I., MacDonald, A., Wright, G., & Hamlin, I. Improving the practical application of the Delphi method in group-based judgment: A six-step prescription for a well-founded and defensible process. Technological Forecasting and Social Change. 2019; 147: 72-82. [DOI: 10.1016/j.techfore.2019.07.002](https://doi.org/10.1016/j.techfore.2019.07.002)

[38] García-Uceda, E. y Murillo-Luna, JL [Application of the Delphi method for the analysis of the determinants of Social Entrepreneurship.]. Ponencia. XXVII Jornadas Hispano_Lusas de Gestión Científica. Localización y dinámicas competitivas en un entorno global. Libro de ACTAS/ Resúmenes. 2017. IBSN: 978-84-16724-37-6; 1 – 4 febrero. Benidorm (Alicante).

[40] Martínez E. The Delphi Technique as a stakeholder consultation strategy for programme evaluation. Revista de Investigación Educativa. 2003: 21(2): 449-163.

[41] García, L., Fernández, S. Procedimiento de aplicación del trabajo creativo en grupo de expertos. Energética, XXIX (2). 2008; 46-50.

[42] Devaney, L., & Henchion, M. (2018). Who is a Delphi ‘expert’? Reflections on a bioeconomy expert selection procedure from Ireland. Futures; 2018; 99: 45-55. DOI: 10.1016/J.FUTURES.2018.03.017.

[45] Boulkedid, R., Abdoul, H., Loustau, M., Sibony, O., & Alberti, C. (2011). Using and Reporting the Delphi Method for Selecting Healthcare Quality Indicators: A Systematic Review. PLoS ONE. 2011;6(6):e20476. doi: 10.1371/journal.pone.00204766.

[50] Landeta, J., y Lertxundi, A. Quality indicators for Delphi studies. Futures & Foresight Science. 2024; 6(1). e172. [DOI: 10.1002/ffo2.172](https://doi.org/10.1002/ffo2.172)

[51] Niederberger, M., & Spranger, J. Delphi Technique in Health Sciences: A Map. Frontiers in public health. 2020; 8: 457. DOI: 10.3389/fpubh.2020.00457.

[52] Skulmoski, G. J., Hartman, F. T., & Krahn, J. The Delphi method for graduate research. Journal of Information Technology Education: Research. 2007; 6(1), 1-21. DOI: 10.28945/199

[53] Niederberger, M., Köberich, S., & DeWiss Network. Coming to consensus: the Delphi technique. European Journal of Cardiovascular Nursing. 2021; 20: 692–695. DOI:10.1093/eurjcn/zvab059

**The team conducting the research: who they are, indicators of their knowledge of the technique and the object of study.**

We have included a more detailed description of the research team's composition in the methodology section. Their specific areas of expertise are highlighted, including labor inclusion of people with disabilities, public policy design, participatory methods such as the Delphi technique, advanced data analysis, social inclusion projects, and extensive practical experience working directly with individuals with disabilities

The following has been added:

Lines: 175-180: The research team conducting this study consists of five researchers with interdisciplinary expertise: a specialist in labor inclusion and public policy with extensive experience in participatory methods such as the Delphi technique, a specialist in business management, a statistician with expertise in data analysis, a professional specializing in social inclusion projects, and a researcher with extensive practical experience working directly with people with disabilities.

**Selection of experts: who carries it out, more explicit selection criteria.**

In the revised methodology, a detailed description of the expert selection process, carried out by the research team, has been added. This process was based on clear and explicit criteria designed to ensure the quality and relevance of the contributions to the Delphi process.

One of the main criteria was a minimum of five years' experience in labor inclusion or public policy, which ensured that the experts had a deep and practical knowledge of the topic. In addition, a balanced representation of key sectors was sought, including the third sector, mainstream business and academia, to incorporate diverse and complementary perspectives.

Likewise, priority was given to the participation of experts who had contributed to relevant publications or projects in the area. This approach not only reinforces the diversity of the panel, but also ensures that the contributions are grounded in meaningful experience and knowledge. These combined criteria strengthen the validity of the study and ensure a robust and representative analysis of the issues addressed.

The following has been added:

Lines:182-204: According to Martínez [40], experts are defined as individuals with extensive knowledge and proven experience in the field, whose predictions are highly reliable. These experts possess the ability to provide well-founded evaluations of specific problems and offer actionable recommendations. In this study, the selection criteria were explicitly defined to include professionals with: a) a minimum of five years of experience in the field of inclusion and disability, b) proven expertise demonstrated through publications, project participation, or policy development, c) representation from diverse sectors, such as academia, the third sector, and conventional companies, to ensure a multidimensional perspective.

The selection process was guided by established frameworks, including the experts´ relationship to the research problem, professional experience, personal attributes relevant to participation in Delphi studies, and domain-specific expertise [41]. To preserve objectivity and minimize bias, all participants contributed anonymously and independently throughout the process, with measures in place to prevent contact between panel members. This approach enhanced the reliability of the data collected and ensured unbiased contributions from each expert [42].

The choice of the ideal number of experts depends on several factors, such as ensuring that it is sufficient, avoiding excessive loss of experts during repeated analysis phases, accessibility to experts, manageable workload, and speed in delivering preliminary results [43]. There are different recommendations, such as that of Landeta [44], who suggests that the appropriate range is between 7 and 30 experts, or that of Boulkedid et al. [45], who establish that the median number of panel members is 17, with an interquartile range of 11 to 31 experts.

**Round 0, open-ended questions: how these questions are defined, literature supporting the content of the selected questions.**

We have significantly expanded the methodology section to describe this process. In particular, we have explained how the design of the open-ended questions was guided by specific methodological recommendations of the Delphi method and informed by key literature on labor inclusion and human resource management.

The change reflects several key aspects that strengthen the methodology of the study. First, the use of open-ended questions has been justified, highlighting their importance in exploratory research such as this. Open-ended questions allow for capturing a diverse range of initial perspectives before consolidating them into a structured questionnaire, thus enriching the understanding of the topic at hand. This approach is aligned with the methodological recommendations of the Delphi method, proposed by authors such as Dalkey and Helmer (1963), Rowe and Wright (1999) and Fernández-Ballesteros (1995), who emphasize the relevance of qualitative inputs to establish a solid conceptual base.

In addition, the rigorous process of question design, based on an exhaustive literature review, has been described. Key sources, such as Peiró and Prieto (2007), Schur et al. (2014) and Lindsay et al. (2018), provided the theoretical basis for defining the scope and content of the questions. For example, questions specific to recruitment and selection processes were designed to explore essential criteria, supported by studies that highlight organizational policies and practices as critical factors in workplace inclusion. This approach ensures that the questions are relevant, clear and aligned with the objectives of the study.

The content of the questions was expanded to include key topics such as skills, job performance, and organizational climate. These areas were informed by the frameworks proposed by Ishikawa (1991) and Aguelo and Coma (2016), which identify these dimensions as critical indicators of job success for people with disabilities. Incorporating these topics ensured that the questions addressed essential factors influencing workplace integration and employee performance.

Additionally, questions related to barriers and strategies for improving job inclusion were included. These were supported by recent research, such as the studies by Gibbons and Salkever (2019) and Lindsay et al. (2018), which explore specific challenges faced by people with disabilities in the labor market. This approach provided a comprehensive perspective on the obstacles to inclusion and highlighted potential pathways for creating more inclusive work environments.

The design of the questionnaire was developed through a clearly defined three-step process. The first step consisted of a comprehensive literature review, which identified key dimensions related to the labor market inclusion of people with disabilities. These dimensions included hiring practices, work culture, and specific barriers to workplace integration. This analysis provided the theoretical basis for the development of the questions.

In the second step, a group of five external experts reviewed the initial questions. These experts, selected for their experience in labor inclusion and qualitative methodologies, assessed the clarity, relevance and appropriateness of the questions in relation to current labor market challenges. This review process ensured that the questions were relevant and aligned with the objectives of the study.

Finally, in the third step, an iterative refinement was carried out. Comments and suggestions provided by the experts were integrated to improve the questions. This process ensured that the questions were open-ended but specific enough to elicit meaningful responses, laying a solid foundation for the subsequent phases of the study.

The connection with the Delphi process has been highlighted as a central aspect of the methodological design. The initial questions not only facilitated the identification of key issues related to labor inclusion, but also served as a solid conceptual basis to guide the subsequent phases of the Delphi process. These exploratory questions allowed for the collection of a wide range of perspectives that enriched the analysis.

In the process phases, the initial inputs were refined and validated through rigorous analysis. This process included thematic coding, which allowed responses to be organized into meaningful categories, and consensus-building among the participating experts. These strategies ensured that the final indicators were representative, relevant and aligned with the objectives of the study.

For explaining this process, the following paragraph have been added:

Lines 226-273: For the first questionnaire corresponding to phase 1, we opted for a design that included open-ended questions to obtain preliminary information on the relevant indicators for evaluating the labor inclusion of people with disabilities. Open-ended questions are particularly suitable for exploratory research as they allow for a broad and diverse range of initial perspectives to be captured before consolidating them into a structured questionnaire. This approach is consistent with the methodological recommendations for the Delphi technique, which emphasize the importance of qualitative input to establish a robust conceptual foundation [34,36,46].

The open-ended questions were developed through a rigorous process based on a comprehensive review of the literature on labor inclusion and human resource management. Key sources, such as Peiró & Prieto [11], Schur et al. [47], and Lindsay et al. [48], provided fundamental insights for defining the scope of the questions. For instance, questions addressing recruitment and selection processes were designed to explore the criteria and aspects critical for evaluating these processes when hiring people with disabilities in conventional companies. An example question was: “What criteria or aspects are important when evaluating the recruitment and selection process of people with disabilities in a conventional company?” These questions were informed by studies that emphasize the significance of organizational policies and practices as pivotal areas for analysis.

Additionally, topics such as skills, job performance, and work environment were included in the questionnaire. These aspects were based on the frameworks proposed by Ishikawa [9] and Aguelo & Coma [8]. Their work highlights these dimensions as key indicators for assessing the success of people with disabilities in the workplace. By incorporating these themes, the questions aimed to gain a comprehensive understanding of the factors influencing the labor inclusion of people with disabilities.

In addition, the content of the questions on potential barriers and strategies for improvement was guided by recent research addressing specific challenges faced by people with disabilities (PwD) in the labor market, such as attitudinal barriers, accessibility issues, and support systems [14,48].

The design process began with Literature Mapping, which involved a systematic review to identify key dimensions of labor inclusion. This review focused on areas such as recruitment practices, workplace culture, and specific barriers to employment. These dimensions provided the basic framework for the development of open-ended questions.

An Expert Review was then carried out. A draft of the questions was carefully examined by a panel of five external experts. These experts, who possessed significant experience in disability inclusion, business management and qualitative research methods, provided critical feedback. This step ensured that the questions were relevant, clear, and aligned with the current challenges and trends in the field of labor inclusion.

Finally, an Iterative Refinement phase was carried out. Feedback from the experts´ review was incorporated to further refine the questions. This process ensured that the questions were open-ended yet sufficiently focused to elicit meaningful and actionable responses. This iterative approach enhanced the overall quality and accuracy of the questionnaire.

By starting with open-ended questions, the study ensured that the resulting indicators were based on diverse and comprehensive perspectives. This approach not only facilitated the identification of key themes but also provided the foundation for the subsequent phases of the Delphi process, where these initial inputs were refined and validated through thematic coding and consensus building.

**Better explanation of how responses are interpreted, grouped, discarded or selected to be incorporated into the closed-ended questionnaire in phase 2.**

This section now clarifies the methodological steps taken to ensure the reliability and relevance of the findings derived from the open-ended questionnaire.

In the revised manuscript, we explain that the analysis followed a systematic, multi-stage process to transform the qualitative input from the experts into measurable indicators. Specifically, all responses were subjected to thematic coding to identify recurring concepts and key ideas. This initial interpretation allowed us to group similar responses into preliminary categories, such as recruitment and selection, job performance, and organizational climate. These categories were refined through consensus among the research team to maintain rigor and objectivity in aligning the data with the study's objectives.

Further, the categories were evaluated for relevance, clarity, and frequency of appearance among experts. Responses that did not align with the study’s objectives or were anecdotal or unclear were systematically excluded. The final selected categories were then transformed into specific items for the closed-ended questionnaire in Phase 2. Each item was carefully crafted to ensure clarity and precision, reflecting the diversity of perspectives captured in the initial responses.

We have also highlighted how these methodological steps are aligned with best practices in Delphi studies, as emphasized in relevant literature (e.g., Niederberger & Spranger, 2020; Rowe & Wright, 1999). This systematic approach ensures the robustness of the data and enhances the reliability of the indicators developed in subsequent phases.

The revised manuscript now includes additional details on how these steps contribute to the validity of the study and how they were designed to balance methodological rigor with the practical considerations of working with a diverse panel of experts. These clarifications address the reviewer's concerns and provide greater transparency regarding the methods used.

The following has been added:

Lines 278-305: A structured semantic analysis was used to interpret and categorize the answers obtained to these questions. This approach was designed to ensure the validity of the dimensions identified, following the recommendations of methodological studies on the Delphi method [29,49,50].

The responses were analyzed qualitatively following a systematic approach of categorization and synthesis [51,52]. This process involved several key steps to ensure the validity and relevance of the findings.

First, all open-ended responses were reviewed to identify key ideas and recurring concepts. Qualitative content analysis through thematic coding was used to structure the information [36]. Secondly, similar ideas were grouped into preliminary categories, such as recruitment and selection, job performance, and work environment. This grouping process was carried out by consensus among the researchers, ensuring rigor and objectivity in the interpretation of the responses [37].

Thirdly, the categories generated were evaluated for relevance, clarity and frequency among experts. Responses that were anecdotal, unclear, or not in line with the study objectives were systematically discarded to maintain methodological rigor [30,32]. Finally, the selected categories were transformed into specific items for the Phase 2 closed-ended questionnaire. Each item was carefully worded to ensure clarity and accuracy, capturing the essence of the insights provided by the experts [34,53].

An Expert Review was then conducted. A draft of the questions was carefully examined by a panel of five external experts with significant experience in disability inclusion and qualitative research methods, who provided critical feedback. This step ensured that the questions were relevant, clear, and aligned with the current challenges and trends in the field of labor inclusion.

This structured process allowed for a robust translation of qualitative insights into measurable indicators, forming the basis for subsequent phases of the study. In addition, this approach ensures that the categories reflect both the diversity of views and alignment with dimensions recognized in previous research on labor inclusion

**The support of a group of 5 new experts is positive, but, in my opinion, it would have been advisable to accompany it with a pilot test of the second questionnaire with another group of experts.**

We recognize that this approach could have brought additional value to the validation process. However, the study design opted for a detailed review by a group of five new independent experts, who evaluated the content of the second questionnaire in terms of clarity, relevance and alignment with the study objectives.

This process made it possible to identify and adjust key elements of the questionnaire prior to its implementation in the next phase. Although the peer review served as a validation mechanism, we acknowledge the limitation of not having conducted a formal pilot test with another group of participants, which could have provided additional information on its practical applicability and understanding by a wider audience.

In the revised version, we have added the following actions from the ongoing project. These include converting the indicators into a scale, conducting a pilot study with a new group of experts, a survey of participants and a statistical analysis of the data for the creation of an assessment to be implemented in different contexts and populations.

The following has been added:

Lines 615-620: The indicators developed in this study can be the basis for the creation of useful tools (which the research team is already working on) to evaluate and promote the inclusion of people with disabilities in mainstream organizations, to design internal inclusion policies that respond to the specific needs of each organization, to conduct accessibility audits and evaluate inclusive practices, as well as to facilitate the monitoring of the impact of inclusion initiatives in the workplace.

Lines 646-651: On the other hand, limitations include the lack of a formal pilot test of the second questionnaire with an independent group of participants. However, the questionnaire was thoroughly reviewed by a group of five new independent experts, who assessed its clarity, relevance, and alignment with the study objectives.

Nevertheless, the scalability of the indicators and the implementation of a pilot study with a new group of experts are among the actions planned for the next phase of the project.

.

**Third questionnaire: were they not given indicators of dispersion (standard deviation or interquartile range? were they not asked to justify their answer if they stayed out of a more or less centred position (between Q1 and Q3, for example)? were they only asked to justify their answer if they changed their opinion? In my opinion, this induces not to change one's position in order not to have to justify one's answer. It encourages a false stability, limits the exchange of reasoning and the ability of the delphi to generate real changes of opinion resulting from the possession of more information and more reflexion, leading to a higher quality and more consensual group opinion. In fact, the third round contributed practically nothing, so the second round would have been sufficient, i.e. a survey rather than a delphi.**

We appreciate the reviewer's comments on the methodological decisions made in the third round of the Delphi study and the handling of divergent responses. In response to these comments, we have added additional details and reflections in the revised version of the manuscript.

In the third round, participants were provided with two key pieces of information for each indicator: the group mean score obtained in the second round and their own individual score from the same phase. This approach sought to encourage reflection on their position in relation to the group consensus and to allow them to adjust their responses if they felt it was necessary. However, no measures of dispersion, such as standard deviation, median or interquartile range, were included in the information provided. This decision was deliberate and was based on the need to avoid cognitive overload for the participants and to keep the focus on their alignment with the group mean, without introducing unnecessary statistical complexity, particularly for those experts without advanced training in statistical analysis. In addition, this strategy sought to preserve the independence of the experts' judgment and to avoid possible biases arising from variability in the group responses.

The following has been added:

Lines 315-335: After compiling the scores from the second round, a third questionnaire was designed with the aim of reaching a statistical consensus within the group of experts. Each participant was provided with two key pieces of information for each of the 52 indicators: (1) the average score assigned by the group in the second round; and (2) their own individual score from the same round. This design allowed participants to reflect on their position relative to the group consensus and to decide whether they wished to adjust their assessment. In cases where participants chose to modify their responses, they were asked to provide a rationale to capture the reasons for their adjustments.

However, no measures of dispersion, such as standard deviation, median, or interquartile range were shared with the participants. This decision intended to avoid cognitive overload and to keep the focus on the fit of individual responses relative to the group mean. Reducing statistical complexity minimized the risk of distraction, particularly for experts with limited experience in advanced statistical analysis. In addition, this approach sought to preserve the independence of the experts' judgment, reducing the influence that within-group variability might have on their assessments.

Although these measures of dispersion were not presented during the process, they were thoroughly analyzed and are included in the results section. This ensures that the variability and distribution of responses are fully considered in the analysis and interpretation of the data. This methodological decision reflects the balance between providing relevant information and maintaining the clarity and accessibility of the Delphi process [30].

We recognize, as the reviewer points out, that the incorporation of dispersion measures could have added valuable context, enriching the interpretation of variability in the responses and promoting more nuanced discussions of the indicators assessed. However, But that information would not be useful for most experts in the field of human resources or labor insertion (they were not academics), as they are generally not familiar with parametric or non-parametric statistics.

We appreciate the reviewer’s thoughtful comments regarding the methodology used in our Delphi study, specifically the decision not to require participants to justify their responses exclusively when changing their opinions. This methodological choice aligns with the fundamental principles of the Delphi method, which is designed to foster thoughtful reflection on the proximity or divergence of expert responses relative to the group average. By encouraging independent evaluation without imposing additional burdens, the process promotes deeper analysis while minimizing potential biases.

Requiring justifications for both unchanged responses and those deviating from a central position (e.g., between Q1 and Q3) could inadvertently introduce biases. Such a requirement might discourage participants from maintaining honest positions to avoid additional workload, potentially inducing false stability. Furthermore, this approach could limit the exchange of reasoning and reduce the Delphi method’s capacity to generate meaningful opinion shifts based on newly acquired information.

While the observed changes between the second and third rounds were minimal, the inclusion of the third round was essential to ensure the stability of expert opinions, a core objective of Delphi studies. Stability and convergence of responses are fundamental indicators of consensus validity in this methodology. The relatively quick convergence of responses in our study reflects the high level of expertise among the panelists.

Quantified Data on Changes and Convergence:

1. **Changes Between the Second and Third Rounds:**
   - A total of 28 out of 48 experts (58.3%) did not modify their initial ratings between the second and third rounds.
   - Across the 40 evaluated items, less than 5% of experts altered their responses, with average variations ranging from -0.1 to +0.21 points across the five dimensions.
2. **Items Affected by Opinion Changes:**
   - Changes were concentrated in a few indicators. For instance:
     - Item SyC-8 ("Evaluate the external support for the person with a disability") showed a mean decrease of -0.1 points.
     - Item CL-5 ("Assess the impact of including people with disabilities on team innovation") demonstrated a mean increase of +0.12 points.
3. **Convergence in the Third Round:**
   - Stability of opinions was not achieved until the third round, justifying its inclusion in the process.
   - Statistical analyses, such as the Wilcoxon test, confirmed that differences between rounds were not statistically significant (p-value > 0.05), supporting the robustness of the achieved consensus.

From our point of view the third round was necessary since it overlooks one of the fundamental principles of the Delphi methodology: achieving opinion stability and validating consensus. Although changes during this phase were minimal, the third round played a critical role in documenting response convergence and ensuring that the results are both representative and statistically robust.

**Analysis of results**

**The six indicators that reflected different group opinions between the group of PWD experts and the group of contracting companies were eliminated. In my opinion these would have been the most interesting indicators to promote interaction and argumentation. In any case, I believe that the argument for incorporating or removing indicators should have been the average importance assigned to each indicator rather than the consensus reached. And if in this case the average importance was low, I think that should be highlighted.**

We recognize that these indicators could have represented valuable opportunities to encourage interaction and argumentation within the Delphi process, contributing to a richer and deeper analysis.

In the methodological design, it was decided to eliminate these indicators after in-depth discussion within the research team due to their lack of significant consensus among the expert groups. The decision was guided by the intention to prioritize the cohesion and practical applicability of the selected indicators. However, we acknowledge the reviewer's point and agree that the lack of consensus could have been approached as an opportunity to foster a more robust dialogue among participants, potentially enriching the Delphi process and broadening the scope of perspectives considered.

In the revised version of the manuscript, we have added a discussion of this limitation and reflected on the decisions made. In addition, we have included information on the average level of importance assigned to these indicators, highlighting whether this score was low or whether there were notable differences between groups. This allows for a more complete interpretation of the results and highlights the reasons behind the methodological decisions.

We also recognize that, in future studies, it would be valuable to adopt a dual approach to indicator selection: considering both the level of consensus and the average importance assigned by participants. This approach would make it possible to preserve potentially controversial indicators that, while not achieving immediate consensus, may stimulate deeper and more thoughtful interactions among expert groups.

The following has been added:

Lines 662-674: While the elimination of six indicators was intended to ensure the consistency and usefulness of the results, we recognize that their exclusion may have limited the opportunity to further explore differences of opinion between the expert groups. These divergent indicators fostered deeper discussion within the research team, which included a deliberation about the potential value of retaining these indicators and the potential risk of diluting the clarity and focus of the final results. However, this process underscored the importance of transparently documenting methodological decisions and highlighted the need for future research to analyze divergent opinions as part of a more comprehensive exploration of labor market inclusion challenges. Although the study design included a carefully selected panel of experts and iterative validation of the indicators during the early phases, the incorporation of additional review by external experts at this stage could have enriched the external validity and applicability of the results.

**It would be interesting to have information on the qualitative contributions made in the open question after each item in the second questionnaire and the experts who modified their opinion. Quantitative data and selection of noteworthy qualitative contributions, if applicable.**

To provide more detailed information on qualitative contributions and modifications in expert opinions, we have expanded the manuscript to include additional details in the results section on these aspects.

We now report that 30% of the experts (14 of 48) adjusted their responses on at least one item between the second and third rounds. The changes were mainly minor adjustments, with average variations ranging from -0.1 to +0.25 points. The items with the highest frequency of changes were Selection and Recruitment (SR8): “Evaluate external support (family/social/institutional) of the person with a disability”; and Work Environment (WE8) “Impact of the incorporation of people with disabilities on team innovation”. In both cases, the modifications were due to discussions about their relevance and to the different interpretations of the experts regarding the importance of these indicators.

In addition, we have highlighted significant qualitative contributions that enriched the interpretation and understanding of the indicators.

These inputs provide context for interpreting the scores and contributed to a better understanding of the relevance of the indicators, which strengthen the manuscript by offering a richer perspective on the process of consensus building and the reasoning behind expert assessments.

The following has been added:

Lines 424-435:  Regarding the qualitative contributions, it is worth mentioning that they provided a valuable context to interpret the results. Some relevant examples are highlighted below: Selection and Recruitment (SR 9): “It is essential to define the characteristics of the position in a clear and accessible manner to avoid initial barriers that may discourage candidates with disabilities.”; Work Itinerary (WI2): “Training itineraries must be adapted not only to the type of disability, but also to the work context and the technological tools available.”; Work Performance (WP10): “Problem solving is a competency that should be fostered in people with disabilities through specific development programs.”; Work Environment (WE3): “Colleagueship can be significantly improved when awareness activities are conducted prior to the incorporation of a person with a disability.”; Socially Responsible Company (SRC5): “The social and cultural benefits of including people with disabilities are difficult to quantify, but critical to strengthening organizational cohesion.”

Lines 469-477: Regarding changes in opinion between round 2 and round 3, 30% of the experts (14 out of 48) adjusted their responses on at least one item between both rounds. The changes were mainly minor, with average variations between -0.1 and +0.25 points.

The items with the highest frequency of change were: Selection and Recruitment (SR8): “Assess external support (family/social/institutional) of the person with disability” and Work Environment (WE5): “Impact of the incorporation of people with disabilities on team innovation”. In the case of SR8, significant adjustments were recorded due to the experts´ interpretation of the importance of this indicator. For WE5, there was a debate about its relevance, which led some experts to modify their scores.

**It would be interesting to accompany the work with an assessment by experts external to the research team of the quality of the development of the Delphi study, the usefulness of its results and the trustworthiness and agreement with them, based, for example, on a brief survey of the panel of participant experts.**

We appreciate the reviewer's suggestion to include an evaluation by experts external to the research team of the quality of the development of the Delphi study, the usefulness of its results, and the reliability of the consensus reached. We consider that this proposal provides significant value for future methodological designs and can contribute to reinforcing the external validity and applicability of the findings.

In the study presented, the Delphi process was developed with the participation of a carefully selected panel of experts and prior validation of the indicators through revisions and adjustments in the early phases of the study. The methodology was designed and executed by a research team with experience in the implementation of this technique (García-Uceda, 2017). However, the research team’s work continues with the design of a scale, relying on another panel of external experts who have so far validated the obtained results.

Likewise, we are confident that the results obtained in this study already reflect its robustness and applicability, supported by the iterative process of consensus and validation carried out with experts in the workplace.

The following has been added:

Lines 675-679: Another limitation is that the present study lacks an independent external evaluation of the quality of the development of the Delphi process. Although the study design included a carefully selected panel of experts and iterative validation of the indicators during the early phases, we recognize that the incorporation of additional review by external experts could have enriched the external validity and applicability of the results.

**Discussion
I believe that the discussion should focus on highlighting what the different experts consider most important for measuring the quality of the labour insertion of pwd, and whether or not they fit with what the academic literature reflects so far, accompanied by reasoned explanations for these potential mismatches, rather than making assertions that are not derived from the results of this study, such as lines 390-392 or 397-405.**

The discussion has been restructured in the revised version of the manuscript to highlight more clearly the overlaps and discrepancies between the results obtained and the existing literature. This approach allows the findings to be placed in a broader academic context, highlighting how the indicators developed reinforce or challenge previously established perspectives in the field of labor inclusion. These comparisons enrich the discussion by providing a more nuanced analysis of the results and their theoretical implications.

In addition, detailed reflections on the practical applications of the indicators have been added, specifying the possible users and contexts in which they could be used. For example, their relevance for companies, third sector organizations, public entities and researchers is mentioned, explaining how these actors can use the indicators to design inclusive strategies, evaluate policies or develop additional studies. This level of specificity reinforces the practical usefulness of the study and its potential impact in different work scenarios.

The discussion explicitly addresses the local setting of the study as a limitation. It is recognized that the results reflect a specific geographical and cultural context, which may restrict their immediate applicability in other settings. To address this limitation, future lines of research have been proposed to validate and adapt the indicators in international contexts and specific sectors. This approach ensures greater relevance and transferability of the findings in subsequent studies.

We agree that the statements in lines 390-392 and 397-405 go beyond the scope of the study’s results and make assertions that are not supported by the data obtained. In light of this, we have removed these statements from the manuscript to ensure that all conclusions and discussions are firmly grounded in the findings of the study. We appreciate the reviewer’s observation, which has helped us improve the rigor and accuracy of the manuscript.

**In line with the suggestions made for the focus of the work, I believe that reflections on what use these indicators might be and for whom, as well as how they should be completed and by whom, should also be included in more detail in this final section.**

We are grateful for the reviewer's comments, which have allowed us to enrich the final section of the manuscript. In the revised version, we have included detailed reflections on several key aspects related to the indicators developed. First, we have highlighted how these indicators can be fundamental tools for assessing and improving the occupational inclusion of people with disabilities in mainstream organizations. For example, the indicators can serve as a basis for designing internal workplace inclusion policies, conducting audits of accessibility and inclusive practices, and monitoring and evaluating the impact of initiatives related to workplace inclusion. These practical applications underscore their importance as strategic tools in various organizational contexts.

In addition, the revised text explicitly identifies the groups that could benefit from the use of these indicators. Companies can use them to design inclusion strategies and evaluate their implementation, ensuring a more systematic and equitable approach to their labor practices. Third-sector organizations can use them as guidelines to promote inclusive practices among collaborating companies, fostering more inclusive partnerships. Public entities could use them to set standards and evaluate labor inclusion programs, ensuring alignment with regulations and social objectives. Finally, researchers could use these indicators as a conceptual framework to carry out comparative or longitudinal studies to expand knowledge in this area.

Finally, we have added specific details on the procedure for applying the indicators, ensuring their adaptability to different contexts. Internal evaluators, such as human resources managers and team leaders, could complete the indicators related to hiring processes, organizational climate and accessibility. It is also recommended to include the perspective of employees through surveys of workers with and without disabilities to capture their perceptions of inclusion and work climate. In some cases, external audits are suggested, in which independent auditors or specialized consultants evaluate compliance with inclusion standards. This comprehensive approach ensures that indicators are effectively implemented and provide valuable information to improve inclusion practices.

**The local character of the work should also be reflected as a limitation, and it would be advisable to explain its possibilities of generalisation to which geographical and sectoral spheres it could reach.**

In the revised version of the manuscript, we have included a specific section in the limitations section that addresses this aspect. It is recognized that the proposed results and indicators are conditioned by the geographical, cultural and sectoral context in which the research was developed, which could influence their direct applicability in other settings.

In addition, we have reflected on the generalizability of the work. Although the indicators were designed in a local context, we consider that their fundamental principles and key dimensions are sufficiently broad to be adapted to other environments, provided that the necessary adjustments are made.

The following paragraphs have been added in the discussion section:

Lines 593-694: The findings of this study reinforce the importance of assessing occupational performance and implementing reasonable accommodations using objective indicators, consistent with Schur et al. [47] and Lindsay et al. [48]. These authors emphasize that effective inclusion goes beyond hiring, requiring fair performance assessments and accommodations that enable full participation. In addition, reasonable accommodations can enhance productivity and work environment by reducing structural barriers. In this regard, the indicators developed in this study offer a valuable tool for measuring and improving inclusive employability, helping companies optimize their inclusion strategies and long-term sustainability. The use of dimensions such as organizational and social impact shows similar findings in research highlighting the benefits of diversity for work environment and innovation [65,69].

However, some results are not consistent with the literature consulted. The low importance given by the experts to indicators related to the average hiring time or the percentage of PwD in selection processes contrasts with studies that highlight these elements as fundamental for inclusion [61]. These discrepancies could be explained by differences in the priorities of the participating expert groups and suggest the need to explore more inclusive approaches.

In addition, the implementation of reasonable accommodations, along with continuous awareness-raising for all employees, stand as essential measures to overcome barriers and stereotypes. Research evidences that reasonable accommodations do not represent a significant burden for companies and instead, contribute to talent retention and improved work environment [70,71,72].

The indicators developed in this study can be the basis for the creation of useful tools (which the research team is already working on) to evaluate and promote the inclusion of people with disabilities in mainstream organizations, to design internal inclusion policies that respond to the specific needs of each organization, to conduct accessibility audits and evaluate inclusive practices, as well as to facilitate the monitoring of the impact of inclusion initiatives in the workplace.

Sustainability of inclusive practices is essential to ensure that initiatives are not limited to the short term, but transform organizational culture in a lasting way. (75,76,77]. The indicators developed in this study seek to foster these structural and lasting changes. In addition to assessing current practices, they have the potential to drive sustainable organizational change that benefits both people with disabilities and overall business performance [78,79].

It is necessary to take into account that the implementation of the indicators requires a collaborative approach involving diverse stakeholders, both within the organization (such as disabled and non-disabled employees, human resource managers and team leaders) and outside (e.g., specialized consultants or independent auditors) [80,81,82,83].

This study has both strengths and limitations. One of the strengths is that it addresses a current and globally relevant issue: the inclusion of people with disabilities (PwD) in the labor market. This topic aligns with international efforts to promote inclusive workplaces and fills a critical gap by providing tools to assess and improve labor inclusion [47,68].

The use of the Delphi methodology is another key strength, as its iterative and consensus-based approach ensures an exhaustive collection of expert input, integrating diverse perspectives and adding credibility and depth to the findings [32,34]. The inclusion in the expert panel of employers and professionals in the field of human resources in conventional companies, disability experts, and managers of employability services, as well as people with disabilities working in the field of human resources within disability-focused organizations, reflects the integration of diverse perspectives and is also a strength of this study.

Finally, the statistical validation of the indicators through factor analysis increases the robustness of the study, providing a solid basis for their practical application in various organizational settings [84].

On the other hand, limitations include the lack of a formal pilot test of the second questionnaire with an independent group of participants. However, the questionnaire was thoroughly reviewed by a group of five new independent experts, who assessed its clarity, relevance, and alignment with the study objectives.

Nevertheless, the scalability of the indicators and the implementation of a pilot study with a new group of experts are among the actions planned for the next phase of the project.

In addition, depending on the type of the worker´s disability, some indicators may not be appropriate and this should be taken into account when developing a measurement scale. Also, the applicability of these indicators may vary according to different organizational contexts and economic sectors, as each industry and type of company has unique characteristics. Another limitation is that some indicators may require specific adaptations to be applicable in certain work environments, such as smaller or less resourced enterprises. Future research could replicate this model in other geographical contexts and sectors to assess its validity and adaptability. Including a broader group of participants, both in terms of industries and regions, would strengthen the robustness and applicability of the indicators.

While the elimination of six indicators was intended to ensure the consistency and usefulness of the results, we recognize that their exclusion may have limited the opportunity to further explore differences of opinion between the expert groups. These divergent indicators fostered deeper discussion within the research team, which included a deliberation about the potential value of retaining these indicators and the potential risk of diluting the clarity and focus of the final results. However, this process underscored the importance of transparently documenting methodological decisions and highlighted the need for future research to analyze divergent opinions as part of a more comprehensive exploration of labor market inclusion challenges. Although the study design included a carefully selected panel of experts and iterative validation of the indicators during the early phases, the incorporation of additional review by external experts at this stage could have enriched the external validity and applicability of the results.

Another limitation is that the present study lacks an independent external evaluation of the quality of the development of the Delphi process. Although the study design included a carefully selected panel of experts and iterative validation of the indicators during the early phases, we recognize that the incorporation of additional review by external experts could have enriched the external validity and applicability of the results. Another aspect is that, although the indicators were statistically validated, they need to be tested in diverse real-world work contexts and on a larger scale to assess their practical applicability and effectiveness.

Finally, a crucial aspect for the labor inclusion of people with disabilities is technological accessibility. We recognize its importance and have identified the need to develop specific indicators to assess the adoption and effectiveness of accessible technologies. For future research, it should be noted that the items related to this may be highly variable over a short period of time.

**Conclusions**

This study has identified 26 key indicators for evaluating the labor inclusion of people with disabilities in conventional companies using the Delphi methodology. Further research is needed to analyze the suitability of these indicators in different contexts and types of disability. In addition, these indicators could serve as a reference for the creation of a scale to assess the labor inclusion of this group of people in conventional companies, thus providing tools that support labor inclusion.

**In sum, it is an interesting work, but in my opinion, it lacks a more focused approach and some weaknesses in its theoretical review and in the development of its fieldwork and the analysis of its results, which should be overcome before its publication.**

As said before, thank you very much for your comments and also for the references provided.

# REVIEWER 2

**Congrats for the good and interesting wok done but little more should be done to take it to next level.**

**Please work on the suggestions given, also mention why you choose those questions for phase 1, in fact the logic behind each question should be supported by literature review.**

We are grateful for the reviewer's comments, which allowed us to strengthen the methodological description of the selection and design of the questions used in phase 1. In the revised version, we have included a detailed explanation of the rationale behind each question, highlighting how these are based on the literature review and the objectives of the study.

The following paragraph have been added:

Lines 226-273: For the first questionnaire corresponding to phase 1, we opted for a design that included open-ended questions to obtain preliminary information on the relevant indicators for evaluating the labor inclusion of people with disabilities. Open-ended questions are particularly suitable for exploratory research as they allow for a broad and diverse range of initial perspectives to be captured before consolidating them into a structured questionnaire. This approach is consistent with the methodological recommendations for the Delphi technique, which emphasize the importance of qualitative input to establish a robust conceptual foundation [34,36,46].

The open-ended questions were developed through a rigorous process based on a comprehensive review of the literature on labor inclusion and human resource management. Key sources, such as Peiró & Prieto [11], Schur et al. [47], and Lindsay et al. [48], provided fundamental insights for defining the scope of the questions. For instance, questions addressing recruitment and selection processes were designed to explore the criteria and aspects critical for evaluating these processes when hiring people with disabilities in conventional companies. An example question was: “What criteria or aspects are important when evaluating the recruitment and selection process of people with disabilities in a conventional company?” These questions were informed by studies that emphasize the significance of organizational policies and practices as pivotal areas for analysis.

Additionally, topics such as skills, job performance, and work environment were included in the questionnaire. These aspects were based on the frameworks proposed by Ishikawa [9] and Aguelo & Coma [8]. Their work highlights these dimensions as key indicators for assessing the success of people with disabilities in the workplace. By incorporating these themes, the questions aimed to gain a comprehensive understanding of the factors influencing the labor inclusion of people with disabilities.

In addition, the content of the questions on potential barriers and strategies for improvement was guided by recent research addressing specific challenges faced by people with disabilities (PwD) in the labor market, such as attitudinal barriers, accessibility issues, and support systems [14,48].

The design process began with Literature Mapping, which involved a systematic review to identify key dimensions of labor inclusion. This review focused on areas such as recruitment practices, workplace culture, and specific barriers to employment. These dimensions provided the basic framework for the development of open-ended questions.

An Expert Review was then carried out. A draft of the questions was carefully examined by a panel of five external experts. These experts, who possessed significant experience in disability inclusion, business management and qualitative research methods, provided critical feedback. This step ensured that the questions were relevant, clear, and aligned with the current challenges and trends in the field of labor inclusion.

Finally, an Iterative Refinement phase was carried out. Feedback from the experts´ review was incorporated to further refine the questions. This process ensured that the questions were open-ended yet sufficiently focused to elicit meaningful and actionable responses. This iterative approach enhanced the overall quality and accuracy of the questionnaire.

By starting with open-ended questions, the study ensured that the resulting indicators were based on diverse and comprehensive perspectives. This approach not only facilitated the identification of key themes but also provided the foundation for the subsequent phases of the Delphi process, where these initial inputs were refined and validated through thematic coding and consensus building.

**This process will enhance the reliability and validity of the questions asked and further strengthen your research and the analysis part too.**

Thank you very much this comment. Steps taken to ensure validity, including expert reviews and iterative feedback, are described in detail. References to methodological studies (e.g., Pozo et al., 2007) have been added.

The following has been added:

Lines 135-164: This study was conceived as an exploratory research, and was developed in three phases between July and November 2023. The Delphi method was chosen due to its recognized ability to structure group communication and encourage iterative reflection among experts in solving complex problems, especially in areas with limited evidence [28,29]. This approach has been widely validated in disciplines such as health and social sciences thanks to its effectiveness in building consensus, identifying discrepancies, and designing robust indicator or policy frameworks [30,31].

According to Okoli & Pawlowski [32], the Delphi method is useful in research that deals with the identification and prioritization of important issues for strategic decision making. It makes it possible to obtain informed and systematic opinions from a group of experts, while preserving their anonymity and guaranteeing the independence of their contributions. Furthermore, Flostrand, Pitt & Bridson [33] emphasize that the Delphi method is an effective tool for investigating complex problems from a multidisciplinary perspective, facilitating the integration of different points of view into a structured framework.

Its iterative structure, together with controlled feedback and statistical aggregation of responses, encourages convergence of opinion and informed reflection, reducing the biases of traditional group dynamics [34,35,36] (Fig 1). In addition, its ability to guarantee anonymity in the responses is essential to avoid hierarchical or social relationships between participants, thus reinforcing the validity and objectivity of the results obtained [32,33].

Although there are other qualitative techniques, such as individual interviews or focus groups, they do not offer the same guarantees of anonymity or the level of structuring that characterizes the Delphi method. Its ability to explore complex problems and arrange solutions through multiple rounds of questionnaires makes it particularly suitable for research in novel or data-limited areas [33,37].

Therefore, this method not only ensures the integration of expertise, but also involves a rigorous and efficient methodology for addressing research questions, allowing the development of a robust indicator framework from the contributions of experts from different disciplines [38,39].

Related Phase 0:

Lines 182-204: According to Martínez [40], experts are defined as individuals with extensive knowledge and proven experience in the field, whose predictions are highly reliable. These experts possess the ability to provide well-founded evaluations of specific problems and offer actionable recommendations. In this study, the selection criteria were explicitly defined to include professionals with: a) a minimum of five years of experience in the field of inclusion and disability, b) proven expertise demonstrated through publications, project participation, or policy development, c) representation from diverse sectors, such as academia, the third sector, and conventional companies, to ensure a multidimensional perspective.

The selection process was guided by established frameworks, including the experts´ relationship to the research problem, professional experience, personal attributes relevant to participation in Delphi studies, and domain-specific expertise [41]. To preserve objectivity and minimize bias, all participants contributed anonymously and independently throughout the process, with measures in place to prevent contact between panel members. This approach enhanced the reliability of the data collected and ensured unbiased contributions from each expert [42].

The choice of the ideal number of experts depends on several factors, such as ensuring that it is sufficient, avoiding excessive loss of experts during repeated analysis phases, accessibility to experts, manageable workload, and speed in delivering preliminary results [43]. There are different recommendations, such as that of Landeta [44], who suggests that the appropriate range is between 7 and 30 experts, or that of Boulkedid et al. [45], who establish that the median number of panel members is 17, with an interquartile range of 11 to 31 experts.

Related to Phase 1:

Lines 225-273: For the first questionnaire corresponding to phase 1, we opted for a design that included open-ended questions to obtain preliminary information on the relevant indicators for evaluating the labor inclusion of people with disabilities. Open-ended questions are particularly suitable for exploratory research as they allow for a broad and diverse range of initial perspectives to be captured before consolidating them into a structured questionnaire. This approach is consistent with the methodological recommendations for the Delphi technique, which emphasize the importance of qualitative input to establish a robust conceptual foundation [34,36,46].

The open-ended questions were developed through a rigorous process based on a comprehensive review of the literature on labor inclusion and human resource management. Key sources, such as Peiró & Prieto [11], Schur et al. [47], and Lindsay et al. [48], provided fundamental insights for defining the scope of the questions. For instance, questions addressing recruitment and selection processes were designed to explore the criteria and aspects critical for evaluating these processes when hiring people with disabilities in conventional companies. An example question was: “What criteria or aspects are important when evaluating the recruitment and selection process of people with disabilities in a conventional company?” These questions were informed by studies that emphasize the significance of organizational policies and practices as pivotal areas for analysis.

Additionally, topics such as skills, job performance, and work environment were included in the questionnaire. These aspects were based on the frameworks proposed by Ishikawa [9] and Aguelo & Coma [8]. Their work highlights these dimensions as key indicators for assessing the success of people with disabilities in the workplace. By incorporating these themes, the questions aimed to gain a comprehensive understanding of the factors influencing the labor inclusion of people with disabilities.

In addition, the content of the questions on potential barriers and strategies for improvement was guided by recent research addressing specific challenges faced by people with disabilities (PwD) in the labor market, such as attitudinal barriers, accessibility issues, and support systems [14,48].

The design process began with Literature Mapping, which involved a systematic review to identify key dimensions of labor inclusion. This review focused on areas such as recruitment practices, workplace culture, and specific barriers to employment. These dimensions provided the basic framework for the development of open-ended questions.

An Expert Review was then carried out. A draft of the questions was carefully examined by a panel of five external experts. These experts, who possessed significant experience in disability inclusion, business management and qualitative research methods, provided critical feedback. This step ensured that the questions were relevant, clear, and aligned with the current challenges and trends in the field of labor inclusion.

Finally, an Iterative Refinement phase was carried out. Feedback from the experts´ review was incorporated to further refine the questions. This process ensured that the questions were open-ended yet sufficiently focused to elicit meaningful and actionable responses. This iterative approach enhanced the overall quality and accuracy of the questionnaire.

By starting with open-ended questions, the study ensured that the resulting indicators were based on diverse and comprehensive perspectives. This approach not only facilitated the identification of key themes but also provided the foundation for the subsequent phases of the Delphi process, where these initial inputs were refined and validated through thematic coding and consensus building.

Lines 278-305: A structured semantic analysis was used to interpret and categorize the answers obtained to these questions. This approach was designed to ensure the validity of the dimensions identified, following the recommendations of methodological studies on the Delphi method [29,49,50].

The responses were analyzed qualitatively following a systematic approach of categorization and synthesis [51,52]. This process involved several key steps to ensure the validity and relevance of the findings.

First, all open-ended responses were reviewed to identify key ideas and recurring concepts. Qualitative content analysis through thematic coding was used to structure the information [36]. Secondly, similar ideas were grouped into preliminary categories, such as recruitment and selection, job performance, and work environment. This grouping process was carried out by consensus among the researchers, ensuring rigor and objectivity in the interpretation of the responses [37].

Thirdly, the categories generated were evaluated for relevance, clarity and frequency among experts. Responses that were anecdotal, unclear, or not in line with the study objectives were systematically discarded to maintain methodological rigor [30,32]. Finally, the selected categories were transformed into specific items for the Phase 2 closed-ended questionnaire. Each item was carefully worded to ensure clarity and accuracy, capturing the essence of the insights provided by the experts [34,53].

An Expert Review was then conducted. A draft of the questions was carefully examined by a panel of five external experts with significant experience in disability inclusion and qualitative research methods, who provided critical feedback. This step ensured that the questions were relevant, clear, and aligned with the current challenges and trends in the field of labor inclusion.

This structured process allowed for a robust translation of qualitative insights into measurable indicators, forming the basis for subsequent phases of the study. In addition, this approach ensures that the categories reflect both the diversity of views and alignment with dimensions recognized in previous research on labor inclusion

Regarding Phase 2:

Lines 315-335: After compiling the scores from the second round, a third questionnaire was designed with the aim of reaching a statistical consensus within the group of experts. Each participant was provided with two key pieces of information for each of the 52 indicators: (1) the average score assigned by the group in the second round; and (2) their own individual score from the same round. This design allowed participants to reflect on their position relative to the group consensus and to decide whether they wished to adjust their assessment. In cases where participants chose to modify their responses, they were asked to provide a rationale to capture the reasons for their adjustments.

However, no measures of dispersion, such as standard deviation, median, or interquartile range were shared with the participants. This decision intended to avoid cognitive overload and to keep the focus on the fit of individual responses relative to the group mean. Reducing statistical complexity minimized the risk of distraction, particularly for experts with limited experience in advanced statistical analysis. In addition, this approach sought to preserve the independence of the experts' judgment, reducing the influence that within-group variability might have on their assessments.

Although these measures of dispersion were not presented during the process, they were thoroughly analyzed and are included in the results section. This ensures that the variability and distribution of responses are fully considered in the analysis and interpretation of the data. This methodological decision reflects the balance between providing relevant information and maintaining the clarity and accessibility of the Delphi process [30].

**While reporting the analysis you can refer the literature for each question, and you can get better insights.**

In the revised article, work has been done to connect the study's findings with the existing literature, highlighting overlaps and discrepancies. For example, we analyze how the priorities identified by the experts in this study reinforce, complement or challenge the results of previous research. This not only adds depth to the analysis but also allows reflection on possible areas for improvement and future research.

**Final part, discussion etc is little weak strengthen it and clear state the takeaway.**

In the revised version, the discussion has been significantly expanded to analyze in greater depth the results obtained in relation to the existing literature. A detailed analysis of the overlaps and discrepancies between the experts' perspectives and previous research findings has been incorporated.

The following paragraphs have been added in the discussion section:

Lines 593-694: The findings of this study reinforce the importance of assessing occupational performance and implementing reasonable accommodations using objective indicators, consistent with Schur et al. [47] and Lindsay et al. [48]. These authors emphasize that effective inclusion goes beyond hiring, requiring fair performance assessments and accommodations that enable full participation. In addition, reasonable accommodations can enhance productivity and work environment by reducing structural barriers. In this regard, the indicators developed in this study offer a valuable tool for measuring and improving inclusive employability, helping companies optimize their inclusion strategies and long-term sustainability. The use of dimensions such as organizational and social impact shows similar findings in research highlighting the benefits of diversity for work environment and innovation [65,69].

However, some results are not consistent with the literature consulted. The low importance given by the experts to indicators related to the average hiring time or the percentage of PwD in selection processes contrasts with studies that highlight these elements as fundamental for inclusion [61]. These discrepancies could be explained by differences in the priorities of the participating expert groups and suggest the need to explore more inclusive approaches.

In addition, the implementation of reasonable accommodations, along with continuous awareness-raising for all employees, stand as essential measures to overcome barriers and stereotypes. Research evidences that reasonable accommodations do not represent a significant burden for companies and instead, contribute to talent retention and improved work environment [70,71,72].

The indicators developed in this study can be the basis for the creation of useful tools (which the research team is already working on) to evaluate and promote the inclusion of people with disabilities in mainstream organizations, to design internal inclusion policies that respond to the specific needs of each organization, to conduct accessibility audits and evaluate inclusive practices, as well as to facilitate the monitoring of the impact of inclusion initiatives in the workplace.

Sustainability of inclusive practices is essential to ensure that initiatives are not limited to the short term, but transform organizational culture in a lasting way. (75,76,77]. The indicators developed in this study seek to foster these structural and lasting changes. In addition to assessing current practices, they have the potential to drive sustainable organizational change that benefits both people with disabilities and overall business performance [78,79].

It is necessary to take into account that the implementation of the indicators requires a collaborative approach involving diverse stakeholders, both within the organization (such as disabled and non-disabled employees, human resource managers and team leaders) and outside (e.g., specialized consultants or independent auditors) [80,81,82,83].

This study has both strengths and limitations. One of the strengths is that it addresses a current and globally relevant issue: the inclusion of people with disabilities (PwD) in the labor market. This topic aligns with international efforts to promote inclusive workplaces and fills a critical gap by providing tools to assess and improve labor inclusion [47,68].

The use of the Delphi methodology is another key strength, as its iterative and consensus-based approach ensures an exhaustive collection of expert input, integrating diverse perspectives and adding credibility and depth to the findings [32,34]. The inclusion in the expert panel of employers and professionals in the field of human resources in conventional companies, disability experts, and managers of employability services, as well as people with disabilities working in the field of human resources within disability-focused organizations, reflects the integration of diverse perspectives and is also a strength of this study.

Finally, the statistical validation of the indicators through factor analysis increases the robustness of the study, providing a solid basis for their practical application in various organizational settings [84].

On the other hand, limitations include the lack of a formal pilot test of the second questionnaire with an independent group of participants. However, the questionnaire was thoroughly reviewed by a group of five new independent experts, who assessed its clarity, relevance, and alignment with the study objectives.

Nevertheless, the scalability of the indicators and the implementation of a pilot study with a new group of experts are among the actions planned for the next phase of the project.

In addition, depending on the type of the worker´s disability, some indicators may not be appropriate and this should be taken into account when developing a measurement scale. Also, the applicability of these indicators may vary according to different organizational contexts and economic sectors, as each industry and type of company has unique characteristics. Another limitation is that some indicators may require specific adaptations to be applicable in certain work environments, such as smaller or less resourced enterprises. Future research could replicate this model in other geographical contexts and sectors to assess its validity and adaptability. Including a broader group of participants, both in terms of industries and regions, would strengthen the robustness and applicability of the indicators.

While the elimination of six indicators was intended to ensure the consistency and usefulness of the results, we recognize that their exclusion may have limited the opportunity to further explore differences of opinion between the expert groups. These divergent indicators fostered deeper discussion within the research team, which included a deliberation about the potential value of retaining these indicators and the potential risk of diluting the clarity and focus of the final results. However, this process underscored the importance of transparently documenting methodological decisions and highlighted the need for future research to analyze divergent opinions as part of a more comprehensive exploration of labor market inclusion challenges. Although the study design included a carefully selected panel of experts and iterative validation of the indicators during the early phases, the incorporation of additional review by external experts at this stage could have enriched the external validity and applicability of the results.

Another limitation is that the present study lacks an independent external evaluation of the quality of the development of the Delphi process. Although the study design included a carefully selected panel of experts and iterative validation of the indicators during the early phases, we recognize that the incorporation of additional review by external experts could have enriched the external validity and applicability of the results. Another aspect is that, although the indicators were statistically validated, they need to be tested in diverse real-world work contexts and on a larger scale to assess their practical applicability and effectiveness.

Finally, a crucial aspect for the labor inclusion of people with disabilities is technological accessibility. We recognize its importance and have identified the need to develop specific indicators to assess the adoption and effectiveness of accessible technologies. For future research, it should be noted that the items related to this may be highly variable over a short period of time.

**Conclusions**

This study has identified 26 key indicators for evaluating the labor inclusion of people with disabilities in conventional companies using the Delphi methodology. Further research is needed to analyze the suitability of these indicators in different contexts and types of disability. In addition, these indicators could serve as a reference for the creation of a scale to assess the labor inclusion of this group of people in conventional companies, thus providing tools that support labor inclusion.

Review of manuscript "INDICATORS TO EVALUATE THE LABOUR INSERTION OF PEOPLE WITH DISABILITIES IN CONVENTIONAL COMPANIES IN SPAIN: A DELPHI STUDY."

This manuscript focuses on developing indicators to evaluate the labor market insertion of people with disabilities (PwD) in conventional companies in Spain. Below is a critique based on various aspects of the study:

Strengths:
      1.    Timely and Relevant Topic: The inclusion of PwD in the labor market is a globally significant issue, and the study aligns with ongoing efforts to promote inclusive workplaces.
      2.    Use of Delphi Methodology: The iterative and consensus-driven Delphi method ensures comprehensive input from experts, lending credibility to the findings.
      3.    Comprehensive Indicator Development: The creation of 26 indicators across four dimensions (Work Performance, Labor Management, Social and Organizational Impact, and Competency Assessment) provides a multi-faceted approach to assessing inclusivity.
      4.    Clear Ethical Standards: Ethical approval and anonymization of data strengthen the study's integrity and adherence to research norms.

      5.    Factor Analysis: Statistical validation of the indicators through factor analysis increases the reliability and applicability of the framework.

Weaknesses:
      1.    Overdependence on Expert Opinion:
While the Delphi method is robust, the study heavily relies on expert opinions, which may not fully capture on-ground realities faced by PwD or employers.
      2.    Sample Size and Composition:
The sample size (48 experts) is relatively small and may limit the generalizability of the findings. Additionally, a higher representation of diverse industries could have provided broader insights.
      3.    Limited Stakeholder Inclusion:
The study focuses on HR experts, disability advocates, and company representatives but does not directly involve PwD, whose perspectives are crucial for a holistic evaluation.
      4.    Possible Bias in Indicator Selection:
Indicators were subjectively categorized and selected, which might introduce biases despite attempts to achieve consensus.
      5.    Insufficient Attention to Implementation:
The study identifies indicators but does not provide actionable strategies or tools for companies to implement and measure these effectively.

Suggestions for Improvement:

      **1.    Include PwD in the Study:
Gathering feedback directly from PwD about their workplace experiences can complement expert insights and ensure the indicators address real challenges.... But may be it cant be done now so please mention this as future research**

We acknowledge the importance of incorporating direct feedback from PwD to complement expert insights. Their lived experiences would provide essential context for validating the relevance and applicability of the proposed indicators. While the current study focused on expert perspectives, we have highlighted in the discussion section that future research should actively involve PwD in the design, evaluation, and validation of indicators. This participatory approach would ensure that the framework addresses real workplace challenges faced by PwD.

The limitations of the study have been extended as previously mentioned.

**2.    Expand the Sample:
Involving more participants from diverse industries and regions could increase the robustness and applicability of the findings.**

We agree that involving a larger and more diverse group of participants across industries and regions could strengthen the robustness and generalizability of the findings. In the limitations section, we have noted the relatively localized scope of the expert panel and proposed that future studies should recruit experts and stakeholders from a broader range of sectors and geographical contexts. This would enhance the applicability of the indicators in diverse workplace environments.

The limitations of the study have been extended as previously mentioned.

      **3.    Practical Guidance:
Offer clear guidelines or a toolkit for companies to adopt the proposed indicators and integrate them into their existing HR frameworks.**

To address this suggestion, we have included additional reflections in the discussion section on the practical applications of the indicators. We propose developing a toolkit or clear guidelines for companies to adopt these indicators and integrate them into their HR frameworks. This toolkit could include specific strategies for implementation, best practices, and examples of success stories from inclusive workplaces.

The following has been added:

Lines 615-630: The indicators developed in this study can be the basis for the creation of useful tools (which the research team is already working on) to evaluate and promote the inclusion of people with disabilities in mainstream organizations, to design internal inclusion policies that respond to the specific needs of each organization, to conduct accessibility audits and evaluate inclusive practices, as well as to facilitate the monitoring of the impact of inclusion initiatives in the workplace.

Sustainability of inclusive practices is essential to ensure that initiatives are not limited to the short term, but transform organizational culture in a lasting way. (75,76,77]. The indicators developed in this study seek to foster these structural and lasting changes. In addition to assessing current practices, they have the potential to drive sustainable organizational change that benefits both people with disabilities and overall business performance [78,79].

It is necessary to take into account that the implementation of the indicators requires a collaborative approach involving diverse stakeholders, both within the organization (such as disabled and non-disabled employees, human resource managers and team leaders) and outside (e.g., specialized consultants or independent auditors) [80,81,82,83].

      **4.    Validation in Real-World Contexts:
Pilot testing the indicators in actual workplaces could strengthen their reliability and utility.**

We recognize the importance of testing the proposed indicators in actual workplace settings to validate their reliability and utility. In the revised manuscript, we have emphasized the need for future research to conduct pilot studies in real-world contexts, allowing for iterative refinement of the indicators and providing evidence of their practical impact. This would bridge the gap between theoretical development and practical application.

The following has been added:

Lines 650-651: Nonetheless, the scalability of the indicators and the implementation of a pilot study with a new group of experts are among the actions planned in the next phase of the project.

The following has been added:

Lines 658-661: Future research could replicate this model in other geographical contexts and sectors to assess its validity and adaptability. Including a broader group of participants, both in terms of industries and regions, would strengthen the robustness and applicability of the indicators.

**5.    Focus on Sustainability:
Investigate how these indicators can contribute to long-term changes in workplace inclusivity rather than short-term compliance or reputation management.**

Sustainability in workplace inclusivity is a crucial aspect that aligns with the long-term goals of this study. In the discussion section, we have added reflections on how these indicators can drive lasting changes in workplace culture and practices, moving beyond short-term compliance or reputation management. Future research could investigate the role of these indicators in fostering sustainable inclusivity and building resilient organizational frameworks.

The following has been added:

Lines 621-630: Sustainability of inclusive practices is essential to ensure that initiatives are not limited to the short term, but transform organizational culture in a lasting way. (75,76,77]. The indicators developed in this study seek to foster these structural and lasting changes. In addition to assessing current practices, they have the potential to drive sustainable organizational change that benefits both people with disabilities and overall business performance [78,79].

It is necessary to take into account that the implementation of the indicators requires a collaborative approach involving diverse stakeholders, both within the organization (such as disabled and non-disabled employees, human resource managers and team leaders) and outside (e.g., specialized consultants or independent auditors) [80,81,82,83].

      **6.    Address Technological Accessibility:
Incorporate specific indicators for the use of accessible technologies, which are pivotal for PwD inclusion in modern workplaces.**

The role of accessible technologies is indeed pivotal for the inclusion of PwD in modern workplaces. While technological accessibility was not explicitly addressed as a separate dimension in this study, we recognize its importance and have proposed its inclusion in future research. In the revised manuscript, we suggest developing specific indicators to evaluate the adoption and effectiveness of accessible technologies in facilitating workplace inclusion.

The following has been added:

Lines 683-687: Finally, a crucial aspect for the labor inclusion of people with disabilities is technological accessibility. We recognize its importance and have identified the need to develop specific indicators to assess the adoption and effectiveness of accessible technologies. For future research, it should be noted that the items related to this may be highly variable over a short period of time.

**This manuscript is a solid contribution to disability inclusion research but could benefit from deeper engagement with stakeholders and real-world validation of the proposed indicators.**

We appreciate the reviewer’s thoughtful and constructive feedback. These recommendations have been integrated into the revised manuscript through expanded reflections in the discussion and limitations sections. Additionally, we have outlined concrete pathways for future research to build on these ideas and enhance the impact of the proposed indicators. Thank you for providing these valuable insights, which have significantly enriched the scope and implications of this study.
