# Supplementary material for: Indicators to evaluate the labor insertion of people with disabilities in conventional companies in Spain: A Delphi study
Source: PLoS One. 2025 May 9;20(5):e0322814. doi: 10.1371/journal.pone.0322814 (PMC12063877; doi:10.1371/journal.pone.0322814)
Supplement: Attachment — Submitted filename: Response_to_Reviewers_(03-2025).docx [file pone.0322814.s004.docx]

Dear PhD. Borba Brum,

First of all, we appreciate the effort and time invested in this manuscript. These are very valuable comments that we are sure, they enhance its quality.

We will proceed to respond point by point to the comments from the reviewers.

**1. On the lack of interaction among experts in the Delphi process**

We understand your concern regarding the lack of interaction among the experts and the possibility that the consensus reached did not reflect a genuine change in opinions. To address this concern, we conducted additional validation through a follow-up survey of the experts who participated in the study. This survey assessed their perception of the clarity of the process, the usefulness of the feedback received, the influence of the group mean, and the need for the third round.

The following has been added:

Lines 357-366: “To complement and validate the results obtained in the main study, a follow-up survey was conducted among the experts who participated in the Delphi process. The objective of this survey was to evaluate the clarity of the process, the usefulness of the feedback received in each round, the impact of the justification of changes, the perception of the need for the third round and the applicability of the indicators developed. The survey consisted of 11 Likert-scale questions, where participants rated each item on a scale from 1 (extremely unclear, not useful, strongly disagree, etc.) to 10 (extremely clear, highly useful, strongly agree, etc.). Additionally, an open-ended section allowed experts to provide qualitative feedback. The survey was answered by 21 experts and the results were analyzed using descriptive statistics and qualitative analysis of open comments.”

The results indicated that the Delphi process was considered clear (M=8.14, SD=1.06) and that the feedback received helped to value the responses (M=7.95, SD=1.43). However, the third round was not perceived as essential (M=3.95, SD=0.97), reinforcing the need to review its impact in future research.

The following has been added:

Lines 559-571: “The results of the validation survey confirmed that most experts considered the Delphi process to be clear (M=8.14, SD=1.06) and that the feedback received was useful for reflecting on their answers (M=7.95, SD=1.43). However, the third round was not perceived as essential (M=3.95, SD=0.97), reinforcing the need to discuss its impact on consensus generation. Regarding the applicability of the indicators in labor inclusion, the experts considered them useful (M=7.86, SD=1.19) and applicable to design labor inclusion strategies (M=8.19, SD=1.40).

**Table 7: Results of the validation survey**

|  | **Mean** | **Median** | **SD** | **IQR** | **Min** | **Max** |
| --- | --- | --- | --- | --- | --- | --- |
| **Regarding the Delphi process used in the study, how did you find it?** | **8.14** | **8** | **1.062** | **1.00** | **6** | **10** |
| **To what extent did the feedback received in each round help you to evaluate your answers?** | **7.95** | **8** | **1.431** | **2.00** | **5** | **10** |
| **In the second round, do you think the information provided (group average and your previous response) was sufficient to reflect on your answers?** | **8.33** | **9** | **1.155** | **1.00** | **6** | **10** |
| **Do you consider that the justification for the changes in the answers may have influenced your decision to modify or maintain your answers?** | **7.19** | **7** | **1.806** | **3.00** | **3** | **10** |
| **To what extent do you believe that the consensus reached reflects real and substantiated expert opinions?** | **8.14** | **9** | **1.352** | **2.00** | **6** | **10** |
| **Do you think the third round was necessary to consolidate the consensus or improve the quality of the responses?** | **3.95** | **4** | **0.973** | **2.00** | **2** | **5** |
| **Do you consider that the indicators resulting from the study are applicable to the evaluation of labor inclusion in conventional companies?** | **7.86** | **8** | **1.195** | **2.00** | **6** | **10** |
| **Do you believe that the results obtained in this study are of practical use in the design of labor inclusion strategies?** | **8.19** | **8** | **1.401** | **2.00** | **5** | **10** |
| **Would you recommend conducting future studies that include validation in real work environments before applying these indicators on a large scale?** | **8.10** | **9** | **1.670** | **2.00** | **4** | **10** |

In addition to quantitative responses, some experts provided additional comments on the Delphi process. While several participants highlighted the value of the method for structuring the indicator assessment, others suggested that more interaction among the experts could have enriched the discussion and consensus process.”

**2**. **On the possibility of a false sense of consensus**

Although the third round was not considered fundamental by some experts, the survey confirmed that the consensus reached reflected informed opinions (M=8.14, SD=1.35). To mitigate this limitation, we have included in the discussion and limitations of the study an explicit acknowledgement of this observation, as well as the need to include more dynamic interactions in future applications of the Delphi method.

The following has been added:

Lines: 662-671: “Although the results of the follow-up survey indicated that the third round of the Delphi process was not perceived as essential by some experts, they also validated the usefulness of the indicators generated. This suggests that, although methodological design can be improved in future studies, the results obtained are robust and relevant. Validation in real workplace settings is recognized as a crucial next step to strengthen the applicability of the indicators.

Qualitative comments from the experts reflect mixed perceptions of the Delphi process. While several participants emphasized the usefulness of the method in identifying key indicators, some suggested that more interaction among experts might have supported a more dynamic consensus. In future research, it would be advisable to promote more interaction among experts.”

Lines: 705-709: “While the Delphi method used in this study effectively structured expert input, it did not facilitate direct interaction among participants, which may have limited opportunities for more in-depth discussion. Additionally, some experts perceived the third round as less essential for refining consensus. However, a follow-up validation survey reinforced the relevance of the indicators developed, confirming their perceived usefulness and applicability in assessing labor inclusión.”

**3. Reference 38, from the authors themselves: Summary of a conference proceedings. It does not allow consultation and should not be included among the relevant methodological references.**

We appreciate your comment on Reference 38, which referred to an abstract of a conference proceedings presented by one of the authors. We understand that this type of reference is not easily accessible or verifiable, so we have proceeded to remove it from the manuscript.

**4. Text introduced in lines 226-273: Unnecessarily repeats some ideas in paragraphs 235-241 with those in paragraph 256-259.**

We appreciate your comment on the repetition of ideas in lines 226-273, 235-241, and 256-259. In response to your comment, we have carefully revised these sections and removed redundant paragraphs to improve the flow and clarity of the manuscript.

The following has been changed:

Lines: 225-227: “Phase 1 applied expert judgment to identify key indicators of inclusive employability. The first questionnaire was designed with open-ended questions to gather preliminary insights on labor inclusion for people with disabilities.”

Lines 233-238: “The development of these questions followed a rigorous process, grounded in a comprehensive literature review on labor inclusion and human resource management. Key sources such as Peiró & Prieto [11], Schur et al. [46], and Lindsay et al. [47], guided the formulation of questions addressing critical aspects of recruitment and selection. For example, one question asked: “What criteria or aspects are important when evaluating the recruitment and selection process of people with disabilities in a conventional company? “

Lines 251-253: “This review focused on areas such as recruitment practices, workplace culture, and specific employment barriers, which provided the basic framework for developing the open-ended questions.”

**5. Text introduced on lines 670-674 is repeated in 676-679.**

The following has been deleted:

“~~Although the study design included a carefully selected panel of experts and iterative validation of the indicators during the early phases, the incorporation of additional review by external experts at this stage could have enriched the external validity and applicability of the results.~~”

We sincerely appreciate your support and your motivation to implement the proposed improvements. Your experience encourages us to take on this challenge in order to strengthen the credibility and impact of our work.

We are convinced that these changes will not only benefit the quality of the article, but have also allowed us to deepen our learning about the application of the Delphi method and its validation. We thank you again for your valuable guidance and for taking the time to provide us with detailed and constructive comments.

Kind regards,

Esperanza García-Uceda, PhD
